# Regulation of cell proliferation and transdifferentiation compensates for ventilator‐induced lung injury mediated by NLRP3 inflammasome activation

**DOI:** 10.1002/iid3.1062

**Published:** 2023-10-25

**Authors:** Huan Liu, Xuepeng Yang, Ge Liu

**Affiliations:** ^1^ Department of Anesthesiology Qilu Hospital of Shandong University Ji'nan China; ^2^ Department of Ophtalmology Jinan Second People's Hospital Ji'nan China; ^3^ Department of Ophtalmology Qilu Hospital of Shandong University Ji'nan China

**Keywords:** cell proliferation, NLRP3 inflammasome, transdifferentiation, VILI

## Abstract

**Background:**

Mechanical ventilation is an important means of respiratory support and treatment for various diseases. However, its use can lead to serious complications, especially ventilator‐induced lung injury (VILI). The mechanisms underlying this disease are complex, but activation of inflammatory signalling pathways results in activation of cytokines and inflammatory mediators, which play key roles in VILI. Recent studies have demonstrated that nod‐like receptor protein 3 (NLRP3) inflammasome activation mediates VILI and also accompanied by cell proliferation and transdifferentiation to compensate for alveolar membrane damage. Type I alveolar epithelial cells (AECs I), which are involved in the formation of the blood‐air barrier, are vulnerable to damage but cannot proliferate by themselves; thus, replacing AECs I relies on type II alveolar epithelial cells (AECs II).

**Objective:**

The review aims to introduce the mechanisms of NLRP3 inflammasome activation and its inhibitors, as well as the mechanisms that regulate cell proliferation and transdifferentiation.

**Methods:**

A large number of relevant literature was searched, then the key content was summarized and figures were also made.

**Results:**

The mechanism of NLRP3 inflammasome activation has been further explored, including but not limited to pathogenic and aseptic inflammatory signals, such as, pathogenic molecular patterns and host‐derived danger‐associated molecular patterns activate toll‐like receptor 4/nuclear factor‐kappaB pathway or reactive oxygen species, cyclic stretch, adenosine triphosphate induce K+ efflux through P2X7, Ca^2+^ inflow, mitochondrial damage, etc, eventually induce NIMA‐related kinase 7/NLRP3 binding and NLRP3 inflammasome activation. Not only that, the review also described in detail the inhibitors of NLRP3 inflammasome. And the mechanisms regulating cell proliferation and transdifferentiation are complex and unclear, including the Wnt/β‐catenin, Yap/Taz, BMP/Smad and Notch signalling pathways.

**Conclusions:**

NLRP3 inflammasome activation mediated VILI, and VILI is alleviated after interfering with its activation, and inflammation and repair exist simultaneously in VILI. Clarifying these mechanisms is expected to provide theoretical guidance for alleviating VILI by inhibiting the inflammatory response and accelerating alveolar epithelial cell regeneration in the early stage.

## Graphical abstract

Nod‐like receptor protein 3 inflammasome activation induces ventilator‐induced lung injury (VILI). But, the inflammatory process of VILI is accompanied by the proliferation and transdifferentiation of alveolar epithelial cells, the significance of this phenomenon is to inhibit the occurrence of VILI in the early stage by compensating for the inflammatory response via repairing damaged alveolar membranes.

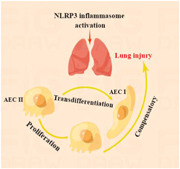

## 1 INTRODUCTION

**Figure 1:**
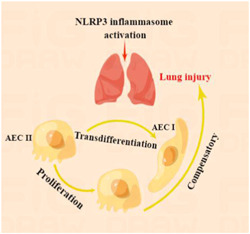
Lung injury mediated by NLRP3 inflammasome activation can be compensated by AEC II proliferation or transdifferentiation into AEC I. Activated NLRP3 inflammasome mediated inflammatory injury in the lung and the body can compensate by AEC II self‐proliferation or transdifferentiation into AEC I. AEC, alveolar epithelial cell; NLRP3, nod‐like receptor protein 3.

**Figure 2:**
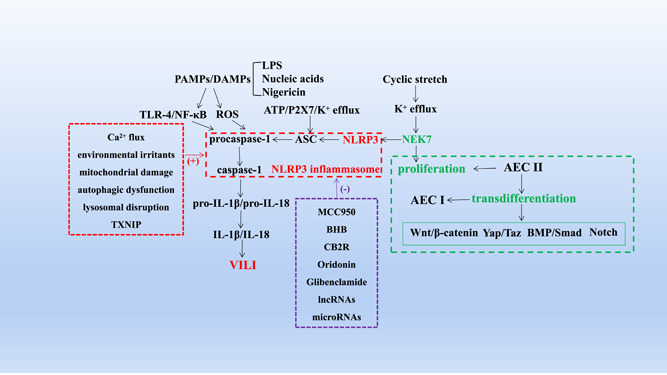
The mechanism of NLRP3 inflammasome activation and its inhibitors as well as the mechanism of regulating cell proliferation and transdifferentiation. PAMPs or DAMPs (LPS, nigericin and nucleic acids, etc.) activate TLR‐4/NF‐κB pathway or ROS, ATP induce K^+^ efflux through P2X7, cyclic stretch activate NEK7 after mediating K^+^ efflux, which activate NLRP3 and induce its assembly with ASC and procaspase‐1 to activate caspase‐1 and promote the maturation and release of IL‐1β and IL‐18. It is worth mentioning that activators of NLRP3 inflammasome also include Ca^2+^ flux, environmental irritants, mitochondrial damage, autophagic dysfunction, lysosomal disruption and TXNIP. While its inhibitors include but are not limited to MCC950, BHB, CB2R, oridonin, glibenclamide, lncRNAs and microRNAs. In addition to participating in NLRP3 inflammasome activation, NEK7 can also regulate cell proliferation, and VILI can be compensated by AEC II proliferation or transdifferentiation into AEC I. Additional pathways involved in the regulation of transdifferentiation mainly include Wnt/β‐catenin, Yap/Taz, BMP/Smad and Notch signalling pathways. AEC, alveolar epithelial cell; ASC, apoptosis‐associated speck‐like protein; ATP, adenosine triphosphate; BHB, β‐hydroxybutyrate; BMP, bone morphogenetic protein; CB2R, cannabinoid receptor 2; DAMP, danger‐associated molecular pattern; lncRNA, long noncoding RNA; LPS, lipopolysaccharide; NEK7, NIMA‐related kinase 7; NF‐κB, Nuclear factor‐kappaB; NLRP3, nod‐like receptor protein 3; PAMP, pathogenic molecular pattern; ROS, reactive oxygen species; Smad, small mothers against decapentaplegic; TLR‐4, toll‐like receptor 4; TXNIP, thioredoxin‐interacting protein; VILI, ventilator‐induced lung injury.

COVID‐19 has spread rapidly worldwide, causing critical illness as well as less severe disease. Mechanical ventilation (MV)‐assisted breathing, which requires endotracheal intubation under general anesthesia, is a critical treatment for acute respiratory distress syndrome (ARDS), acute lung injury, shock, and sepsis.^1^, ^2^, ^3^ MV can significantly improve patients' respiratory function and increase oxygenation to save their lives. However, improper use of MV and inappropriate parameters often lead to oxygenation dysfunction and pulmonary edema, named ventilator‐induced lung injury (VILI). VILI has an estimated prevalence of 10%–65% and a case fatality rate of 13%–55%.^4^, ^5^, ^6^, ^7^ The pathological changes associated with VILI include disruption of alveolar structure, thickening of the pulmonary septum, alveolar hemorrhage, infiltration of neutrophils and macrophages and the formation of transparent membranes.^8^, ^9^ However, the clinical manifestations of VILI are nonspecific and mainly include refractory hypoxemia, pneumothorax, mediastinal emphysema, and increased ventilator pressure. VILI cannot only increase the mortality of patients with existing lung diseases but also affect healthy lungs and other organs by mediating systemic inflammatory reactions.^10^ VILI increases the mortality of patients receiving ventilator treatment, prolongs the length of hospital stay and increases healthcare costs, seriously threatening the life and economic well‐being of patients; thus, it has become a challenging hot topic that must be urgently addressed^7^ (Figures 1 and 2).

Barotrauma, volutrauma, atelectrauma, and biotrauma are the main mechanisms leading to VILI.^5^ When inflammatory damage occurs, barotrauma, volutrauma, atelectrauma can cause biotrauma, and together, they ultimately lead to the development of VILI.^11^ The development and optimization of various ventilation methods, such as appropriate positive end‐expiratory pressure, lung reexpansion and permissible high CO_2_ protective lung ventilation measures, have led to preliminary progress in the prevention and treatment of mechanical injuries.^12^, ^13^, ^14^ However, efforts to prevent biotrauma have not been effective due to the complexity of inflammatory signalling pathways and their cross‐talk. Therefore, there is an urgent need to clarify the pathogenesis of VILI and find effective prevention and treatment measures.

Previous research by our team members revealed that cyclic stretch leads to the degradation and mislocalization of cell junction proteins and increased alveolar capillary permeability, inducing pulmonary edema and thus lung injury, and that activation of inflammatory signalling pathways mediates these processes.^15^, ^16^ MV activates inflammatory signalling pathways to cause the release of inflammatory mediators that not only directly damage lung tissue but also enter the lung by increasing the number of inflammatory cells, promoting the activation and release of cytokines and inflammatory mediators, and the ability of these inflammatory mediators to damage lung tissue should not be underestimated.^8^, ^9^, ^17^ Fortunately, numerous studies have reported that inhibiting inflammation attenuates VILI in preclinical models.^18^, ^19^, ^20^ However, inflammation represents the defensive response of vascular tissue to deleterious factors. During the process of inflammation, damaging factors directly or indirectly cause the destruction of tissues and cells; however, these damaging factors are surrounded and eliminated through inflammatory hyperemia and exudation, and cell regeneration allows damaged tissue to be repaired. Therefore, inflammation has dual injurious and beneficial effects; in the early stage, damaged alveoli can be repaired by cell proliferation and transdifferentiation.

Transdifferentiation, which is essential for repairing cell and tissue damage, is the transformation of a differentiated cell into another normally differentiated cell. Alveolar epithelial cells (AECs) include AECs I and AECs II. AECs I occupy more than 95% of the alveolar surface area and are large flat cells that participate in gas exchange by forming the gas–blood barrier.^21^, ^22^ They cannot proliferate but can be replaced by AECs II proliferation after injury.^23^, ^24^ On the other hand, AECs II occupy 2%–5% of the alveolar surface area and are cubic in shape. They secrete pulmonary surfactants, which can reduce alveolar surface tension. Upon injury, AECs II can generate more AECs II through mitosis or transdifferentiate into AECs I. However, as the inflammation‐immune response expands, AECs are extensively damaged, leading to alveolar collapse. If the alveolar exudate is effectively cleared in time, normal pulmonary anatomy can be restored through self‐repair, that is, through AECs II proliferation and transdifferentiation. If the damage persists, failure of self‐repair processes leads to fibroblast proliferation, massive extracellular matrix aggregation, and dense scar formation due to alveolar collapse, leading to pulmonary fibrosis, which results in persistent impairment of alveolar gas exchange and ultimately the development of VILI.^25^

Previous studies have revealed that MV activates the inflammatory signalling pathway, leading to pulmonary edema and lung injury.^15^, ^16^ The role of inflammation in the pathogenesis of VILI cannot be ignored. In addition to the activation and release of inflammatory mediators and cytokines, the inflammasome plays a key role in VILI. The inflammasomes, located in the cytoplasm, is a multiprotein complex consisting of pattern recognition receptors, which are intracellular signalling platforms that regulate the expression and activation of inflammatory factors. The activated inflammasome is involved in the regulation of the immune response by recognizing different types of pathogens and danger signals and mediates various inflammation‐related metabolic and immune diseases.^26^ The main inflammasomes are the IPAF, nod‐like receptor protein 1 (NLRP1), absent in melanoma 2, leucine‐rich repeat and PYD containing 7, and NLRP3 inflammasomes; among them, the NLRP3 inflammasome has been a hot topic in recent years. It cannot only identify pathogenic molecular patterns (PAMPs) or danger‐associated molecular patterns (DAMPs) but also promote the maturation and release of IL‐1β and IL‐18 after recruitment and activation of procaspase‐1. This review will deeply explore the regulatory mechanisms by which cell proliferation and transdifferentiation compensate for VILI caused by NLRP3 inflammasome activation, providing new theoretical guidance for the clinical prevention of VILI by inhibiting the inflammatory response and promoting cell proliferation and transdifferentiation.

## 2 NLRP3 INFLAMMASOME ACTIVATION MEDIATED VILI

### 2.1 Mechanism of NLRP3 inflammasome activation

The NLRP3 inflammasome, which comprises nucleotide‐binding oligomers, is composed of NLRP3, apoptosis‐associated speck‐like protein (ASC) and cysteine‐aspartic protease 1 (caspase‐1).^27^, ^28^ NLRP3 is known to be activated indirectly by numerous pathogenic and aseptic inflammatory signals, such as lipopolysaccharide (LPS), nucleic acids, nigericin, adenosine triphosphate (ATP), uric acid crystals, potassium efflux, and reactive oxygen species (ROS).^29^, ^30^, ^31^, ^32^, ^33^ The above stimuli lead to the activation of NLRP3, which then binds to the adaptor protein ASC by a homotypic interaction of pyrin domain. Immediately thereafter, ASC undergoes oligomerization and recruits and activates caspase‐1. Finally, the IL‐1β and IL‐18 precursors are processed into their active forms.^34^ Clarifying the mechanisms underlying NLRP3 inflammasome activation can provide guidance for inhibiting the inflammatory response to alleviate VILI.

Kuipers et al. first revealed that the NLRP3 inflammasome signalling pathway is involved in MV‐induced inflammatory responses.^35^ They showed that NLRP3, ASC, and IL‐1β were all upregulated and that caspase‐1 was activated by MV; moreover, VILI was attenuated not only in NLRP3 and ASC knockout(KO) mice but also by inhibition of the IL‐1β and NLRP3 inflammasome pathways. In fact, there are two essential pathways required for IL‐1β activation. One is the toll‐like receptor 4 (TLR‐4)/NF‐κB pathway, Vaneker et al.^36^ confirmed the role of the TLR‐4 pathway in VILI, showing that IL‐1β secretion was reduced in a VILI model after TLR‐4 inhibition. The second step involved the activation of the NLRP3 inflammasome by PAMPs or DAMPs, including the assembly of NLRP3, ASC, and procaspase‐1 into a complex; the transformation of procaspase‐1 into active caspase‐1; and the production and secretion of mature IL‐1β and IL‐18. We previously also illustrated that mechanical force activated the NLRP3 inflammasome, induced the increased secretion of IL‐1β, decreased the expression and distribution of cell junction proteins and the mitochondrial membrane potential, and exacerbated pulmonary edema and lung injury, but the degree of these detrimental changes was reduced and lung injury was alleviated after inhibiting NLRP3 inflammasome activation.^8^ Then, we explored the upstream mechanism by which NLRP3 inflammasome activation is regulated and found that the binding of NIMA‐related kinase 7 (NEK7) and NLRP3 was the core step in this process. Interfering with NEK7/NLRP3 binding through either direct or indirect pathways effectively inhibited NLRP3 inflammasome activation and alleviated VILI.^9^ In addition, some scholars have demonstrated the key role of NEK7 in regulating NLRP3 inflammasome activation in other disease models. This suggests that timely interference with NLRP3 inflammasome activation is expected to inhibit the inflammatory response and alleviate VILI.

One mechanism by which the NLRP3 inflammasome is activated was briefly described above, and we introduce other mechanisms below. Extracellular ATP acts as an NLRP3 agonist to induce K^+^ efflux through P2X7, triggering NLRP3 inflammasome assembly and activation; in this process, K^+^ efflux is a major activator of NLRP3, while extracellular ATP is the major trigger of IL‐1β secretion.^37^ Other studies have shown that intracellular and endoplasmic reticulum Ca^2+^ are also involved in the assembly and activation of the NLRP3 inflammasome.^38^ The PAMPs and DAMPs mentioned above trigger the production of ROS and then induce the assembly and activation of the NLRP3 inflammasome.^39^ When environmental irritants (such as silica, asbestos, amyloid‐β, and alum) form crystalline or particulate structures after being engulfed by phagocytes, they can also trigger NLRP3 inflammasome activation.^40^, ^41^, ^42^, ^43^ This causes lysosomal rupture and the release of lysosomal contents mediated by cathepsin B.^44^ Additional mechanisms, including mitochondrial Ca^2+^ overload, induce mitochondrial damage or dysfunction, autophagic dysfunction, lysosomal disruption, and activation of thioredoxin‐interacting protein.^45^, ^46^ Additionally, it was previously demonstrated that NEK7/NLRP3 binding is a key trigger for subsequent steps in the assembly and activation of the NLRP3 inflammasome induced by the above factors.^47^, ^48^

### 2.2 Inhibitors of NLRP3 inflammasome activation

We have elaborated the mechanisms underlying the activation of the NLRP3 inflammasome and now discuss NLRP3 inflammasome inhibitors that were validated in a preclinical model. Previous studies have shown that MCC950, a diarylsulfonylurea‐containing compound, specifically acts on the NLRP3 inflammasome; it was discovered to inhibit NLRP3‐induced ASC oligomerization and the secretion of IL‐1β and IL‐18. It was later verified in a mouse model that MCC950 ameliorates LPS‐induced lung inflammation.^49^ The ketone metabolite β‐hydroxybutyrate (BHB) was discovered to block NLRP3 inflammasome activation by inhibiting NLRP3‐induced ASC oligomerization and caspase‐1‐mediated IL‐1β production.^50^, ^51^ Moreover, BHB inhibits K^+^ efflux from macrophages. In addition, autophagy has been reported to interfere with NLRP3 inflammasome activation. For example, resveratrol has been shown to inhibit NLRP3 inflammasome activation by mediating autophagy, which is a self‐protective catabolic pathway involving lysosomes, thereby inhibiting mitochondrial damage.^52^ Similar to resveratrol, arglabin and cannabinoid receptor 2 (CB2R) can also induce autophagy and interfere with NLRP3 inflammasome activation.^53^ In addition, oridonin and glibenclamide can exert anti‐inflammatory effects. Oridonin interferes with NLRP3 inflammasome activation by blocking the binding of NEK7 to NLRP3.^54^ Glibenclamide, which is an ATP‐sensitive K^+^ channel inhibitor, has been widely used to treat type II diabetes. Glibenclamide inhibits NLRP3 inflammasome activation by inhibiting potassium efflux and then interfering with the binding of NEK7 and NLRP3.^9^, ^55^ Long noncoding RNAs (lncRNAs) and microRNAs have also been implicated in NLRP3 inflammasome activation. Lnc‐EST12 reduces the expression of IL‐1β and IL‐6 and suppresses NLRP3 inflammasome activation. lncRNA NEAT1 ameliorates LPS‐induced inflammation by activating autophagy and inhibiting NLRP3 inflammasome activation.^56^ MiR‐20a‐5p targets NLRP3 and negatively regulates its expression, but its expression is modulated by lncRNA PVT1.^57^ Thus, the effects of NLRP3 inhibitors on the activation of the NLRP3 inflammasome have been tested in different preclinical models. Previously, we briefly described the mechanisms underlying the activation of the NLRP3 inflammasome and its subsequent effects. We speculate that the abovementioned inhibitors could have a similar effect on VILI as interventions that prevent NLRP3 inflammasome activation, reducing the degree of lung injury. This has also been reported in the literature.^58^

## 3 THE MECHANISMS OF REGULATING CELL PROLIFERATION AND TRANSDIFFERENTIATION

Our team focused on the mechanism by which inflammation causes VILI and found that NEK7 is key for the activation of the NLRP3 inflammasome regardless of the activation pathway. While NEK7 is also involved in regulating mitosis, NLRP3 activation, and mitosis are mutually exclusive events mediated by NEK7.^59^ NEK7, located in the centrosome, is one of the smallest members of the NEK kinase family, is widely expressed in a variety of tissues, is closely related to the formation of the mitotic spindle and the separation of the cytoplasm, and has an indispensable role in regulating the cell cycle.^60^ A study found that deletion of NEK7 arrests cells in metaphase and causes apoptosis^61^; under normal growth conditions, NEK7 activity and levels are low, preventing it from activating the NLRP3 inflammasome and regulating the cell cycle, but it may act as a bidirectional switch.^59^

Once homeostasis is disrupted, NEK7 overexpression induces the production of abnormal cells or inflammatory responses, which triggers various diseases. This finding made us ask whether cell proliferation and transdifferentiation is involved in VILI caused by inflammation. We found that lung injury was accompanied by alveolar self‐repair in VILI. In other words, the damaged alveolar membrane can be repaired by AECs II proliferation and transdifferentiation into AECs I. Our results showed that after cyclic stretching of AECs II, their shape was changed from square to scalar, and they lacked their characteristic lamellar bodies. In addition, the expression of the AECs II marker protein SP‐C decreased, and the expression of the AECs I marker protein caveolin‐1 increased. What are the mechanisms that regulate these phenomena? While the answer to this question is not clear, the evidence presented below may provide helpful clues.

### 3.1 Wnt/β‐catenin signalling pathway

The Wnt gene was identified in a mouse breast cancer model in 1992, and Wnt family members regulate cell proliferation and differentiation during embryonic development. The Wnt signalling pathways include canonical and noncanonical pathways, and the difference between these pathways is whether they depend on β‐catenin. After activating the Wnt/β‐catenin signalling pathway, Wnt protein binds frizzled and low‐density lipoprotein receptor‐related protein to form a complex; this causes active β‐catenin levels to increase and prevents β‐catenin from being phosphorylated and degraded, allowing it to be subsequently transported to the nucleus.^62^ Lymphoid enhancer factor and T‐cell factor form a complex with β‐catenin and enter the nucleus, where they promote the expression of downstream target genes and regulate cell proliferation and differentiation.

Jia Xianxian demonstrated that the expression of Wnt3a was downregulated in the lungs of rats exposed to hyperoxia and that the expression of SP‐C and AQP5 can also be regulated by altering Wnt3a expression.^63^ Importantly, Wnt3a regulated AECs II proliferation and differentiation into AECs I in a model of hyperoxia‑induced bronchopulmonary dysplasia by inducing the nuclear translocation of β‑catenin. However, this pathway can also be regulated by Yes‐associated protein (YAP). The study showed that YAP positively regulated Wnt/β‐catenin signalling by increasing the nuclear translocation of β‑catenin to promote AECs II transdifferentiation into AECs I.

### 3.2 Yap/Taz signalling

Hippo is a Ste20‐like kinase first discovered in Drosophila, and the Hippo signalling pathway includes conserved kinases that play vital regulatory roles in embryonic development, cell proliferation and differentiation, and tissue repair. YAP/TAZ is the most critical molecule in the Hippo pathway, and alterations in its expression and localization may be involved in the regulation of cell proliferation and differentiation. Phosphorylated YAP is located in the cytoplasm, whereas dephosphorylated YAP is activated and translocates to the nucleus; activated YAP together with other transcription factors can promote cell proliferation and differentiation, accelerating repair after lung injury. YAP/TAZ deletion in AECs II impairs AECs regeneration and induces prolonged fibrotic lesions during bacterial pneumonia.^64^

Moreover, stimulation of YAP activity promotes lung inflammation resolution and accelerates lung recovery from injury, and inhibition of YAP activity suppresses epithelial cell regeneration. Therefore, YAP may protect against lung injury through a variety of mechanisms by inhibiting inflammation in the injury phase and promoting lung repair and inflammation resolution in the repair phase.^24^ Other studies have found that human umbilical cord‐derived mesenchymal stem cells (hUC‐MSCs) have immunomodulatory and regenerative potential, and hUC‐MSCs ameliorate lung injury in ARDS and regulate YAP to facilitate AECs II differentiation into AECs I by decreasing pro‐SPC expression while increasing podoplanin (T1α) expression.^65^ It was also reported that verteporfin, a YAP inhibitor, reduces pro‐SPC expression but increases T1α expression. However, in addition to epithelial cell regeneration, epithelial repair is facilitated by mutual communication between activated fibroblasts. Increasing evidence indicates that YAP/TAZ signalling is involved in the pathophysiology of fibrosis, and abnormal activation of YAP/TAZ has been reported in epithelial cells, fibroblasts and myofibroblasts. The mechanism by which YAP/TAZ is involved in pulmonary fibrosis is discussed below.

### 3.3 The bone morphogenetic protein (BMP)/small mothers against decapentaplegic (Smad) pathway

BMPs participate in the processes of bone organogenesis, neurogenesis, embryogenesis, tissue homeostasis, regeneration, and iron metabolism. Smad proteins are intracellular proteins that transduce extracellular signals from transforming growth factor β ligands to the nucleus, where they activate downstream gene transcription.^66^ BMPs bind to receptors at the cell membrane and activate receptor kinases, which phosphorylate Smad l/5/8 after receptor activation, form a complex with Smad 4 and enter the nucleus, thus regulating the transcription of BMP target genes.^67^ The BMP/Smad signalling pathway regulates stem cell differentiation, cell proliferation, migration, apoptosis, and embryonic development, and receptor endocytosis, dephosphorylation of Smad l, and the degradation of signalling molecules are involved in regulating this pathway. This review discusses the regulatory role of this pathway in AEC proliferation and transdifferentiation and the underlying mechanism.

BMP signalling may affect transdifferentiation by regulating the expression of matrix proteins, and the role of the BMP signalling pathway in maintaining the AEC II phenotype and promoting the differentiation of AECs II into AECs I under exogenous stimulation should be considered.^66^ A remarkable study of the regulatory effect of BMP/Smad signalling on the proliferation and differentiation of alveolar stem cells reported that the BMP signalling pathway was activated in both AECs II and AECs I in the homeostatic phase, while BMP signalling was decreased during AECs II proliferation and increased during AECs I differentiation. Interestingly, the study demonstrated that BMP signalling inhibited AECs II proliferation and promoted the transdifferentiation of AECs II into AECs I. Overall, BMP signalling in AECs II maintains the quiescence of these cells during homeostasis; during regeneration, inactivation of the BMP signalling pathway promotes AECs II proliferation, while reactivation of the BMP signalling pathway promotes AECs I differentiation. These studies indicate that we need to further clarify the mechanism by which BMP signalling promotes AECs II differentiation to AECs I and whether the BMP signalling pathway interacts with other signalling pathways that influence self‐renewal and differentiation. As mentioned above, there is convincing evidence that the influence of regulatory pathways on AECs II proliferation and differentiation into AECs I can be increased or decreased in response to injury.^68^ For instance, BMP‐2 stimulation was used to enhance bone‐tendon integration in vitro, suggesting that it stimulates the transdifferentiation process in the interface and fibroblast region.^69^ Therefore, the ultimate goal is not to confirm that this pathway is involved in the regulation of cell proliferation and differentiation but to elucidate the specific underlying mechanism, specifically in various areas throughout the body, whose internal regulatory mechanisms are intricate.

### 3.4 Notch signalling

The Notch signalling pathway is a highly conserved signalling pathway in multicellular organisms. It mediates cell regulation by participating in cell–cell interactions and is controls cell growth, cell proliferation, cell differentiation, cell fate, and tissue differentiation during early development. In mammals, there are four distinct Notch receptors: Notch 1, Notch 2, Notch 3, and Notch 4. The results of several studies indicated that the Notch signalling pathway participates not only in regulating lung development but also in mediating the development of lung diseases.^70^ Delta‐like 1 homolog (Dlk1), a noncanonical Notch ligand, plays multiple roles in cell proliferation and differentiation. Interestingly, Dlk1 may act as a checkpoint to slow proliferation while driving cells toward differentiation, thereby causing the cell/organ to enter a state of growth and hypertrophy.^71^ High‐throughput sequencing of transdifferentiating AECs II revealed the differential expression of numerous genes, with reduced expression of Dlk1 and increased expression of another ligand, Jagged1, which is involved in the Notch pathway.^72^ In addition, different ligands of the Notch pathway have different effects. The results indicated that Dlk1 promoted AECs II proliferation and suppressed cell transdifferentiation, while Jagged1 treatment suppressed AECs II proliferation and promoted AECs II transdifferentiation into AECs I. Additional studies found that Notch 1 expression was increased and Jagged1 expression was low or Jagged1 was absent. The number of AECs II increased, and the rate of transdifferentiation slowed.^73^ Studies have also reported that abnormal expression of Notch 1 is involved in the pathogenesis of lung injury, hyperoxia decreases the expression of Notch 1, and downregulation of Notch 1 can regulate the proliferation and transdifferentiation of AECs II. In brief, the Notch signalling pathway is activated during the proliferative phase of alveolar regeneration but is later deactivated due to upregulation of Dlk1, promoting the transdifferentiation of AECs II into AECs I. The upregulation of Dlk1 is the key to inducing cell differentiation, but it is unclear how its expression is regulated. Clarifying the upstream factors that regulate Dlk1 expression will precisely elucidate how AECs II transdifferentiate into AECs I.

## 4 IN SUMMARY

Mechanical injury and biological injury are the main mechanisms leading to VILI. After a series of explorations, researchers proposed many optimized measures for treating VILI, including low tidal volume MV, PEEP, and prone position MV. Although some progress has been made, VILI cannot be effectively alleviated by these ventilation methods due to the complexity and diversity of the signalling pathways leading to biological injury.^74^ Interestingly, subsequent studies showed that NLRP3 inflammasome activation mediates VILI and that TLR‐4/Nuclear factor‐kappaB (NF‐κB) pathway activation and PAMP‐ or DAMP‐mediated activation of caspase‐1 induces the assembly of NLRP3, ASC and procaspase‐1 to activate the NLRP3 inflammasome and then mediate IL‐1β and IL‐18 activation and secretion. NLRP3 activators include but are not limited to LPS, nucleic acids, melanocytes, ATP, uric acid crystals, potassium, and ROS.^75^ Activation of the NLRP3 inflammasome induces the activation of inflammatory mediators and cytokines, which cause degradation and mislocalization of cell junction proteins and increases alveolar capillary permeability, leading to pulmonary edema and lung injury in VILI. This indicates that VILI can be alleviated by inhibiting NLRP3 inflammasome activation. In addition, MCC950, BHB, CB2R, oridonin and glibenclamide can all act as inhibitors of NLRP3 inflammasome activation, and the role of lncRNAs and microRNAs in NLRP3 inflammasome activation cannot be ignored. The inhibitors of the NLRP3 inflammasome mentioned above interfere with various steps of NLRP3 inflammasome assembly, and it was shown that NEK7 is key for NLRP3 inflammasome activation. Surprisingly, the regulatory effects of NEK7 on mitosis and NLRP3 inflammasome activation are mutually exclusive.^59^ However, the role of NEK7 in inhibiting inflammation and promoting mitosis during lung injury is unclear. Moreover, when lung injury occurs, injurious stimuli such as inflammatory stimuli activate the inflammatory pathway in vivo, leading to a cascade that amplifies the inflammatory responses to induce lung injury. Furthermore, AECs II proliferation and transdifferentiation into AECs I were observed to compensate for the damaged alveolar membrane.

According to numerous studies, the Wnt/β‐catenin, Yap/Taz, BMP/Smad, and Notch signalling pathways are all involved in regulating the differentiation of AECs II to AECs I, and cross‐regulation occurs among these pathways during alveolar regeneration. As previously reported, YAP positively regulates Wnt/β‐catenin signalling by increasing the nuclear translocation of β‑catenin to promote AECs II transdifferentiation into AECs I.^63^ YAP also suppresses inflammation during lung injury and promotes repair and inflammation resolution in the repair stage.^24^ In addition, Dlk1 accelerates AECs II proliferation and inhibits AECs II transdifferentiation, while Jagged1 suppresses AECs II proliferation and promotes AECs II transdifferentiation to AECs I via the Notch signalling pathway.^72^ Interestingly, BMP signalling may affect transdifferentiation by regulating the expression of matrix proteins.^66^ These findings reveal the mechanism underlying AECs II transdifferentiation. However, the upstream mechanism by which the above pathways are activated remains unclear, and the effect of AECs II proliferation and transdifferentiation in compensating for alveolar membrane damage in lung injury models is insufficient. The reason for this is the difficulty in distinguishing the differentiation stages in in vivo regeneration models and the difficulty in accurately elucidating the specific steps of AECs II transdifferentiation into AECs I. Moreover, it is impossible to accurately evaluate the inhibitory effect of gene KO or drug intervention on cell proliferation and transdifferentiation. Despite these difficulties, the exploration of this mechanism is valuable. It is still possible to evaluate the proliferation and transdifferentiation of AECs II by assessing the expression and localization of AECs II‐specific marker proteins such as SP‐C, SP‐D, and AECs I marker proteins AQP5 and caveolin‐1, as well as changes in lamellar bodies in AECs II.^76^

In the repair stage of lung injury, monocyte‐derived macrophages and fibroblasts seem to be involved in the process of AECs II transdifferentiation, but AECs II transdifferentiation can lead to failed repair and eventually to pulmonary fibrosis. Macrophages can be divided into classically activated (M1) and alternatively activated (M2) macrophages. M1 macrophages are involved in the pro‐inflammatory response, while M2 macrophages are closely linked to the anti‐inflammatory response.^77^ Macrophages recruited to the epithelium in response to epithelial cell damage and macrophages residing in the epithelium may drive epithelial cell proliferation by producing Wnt ligands, and in a coculture system, it was shown that macrophages can also directly promote the proliferation of AECs II.^78^ M2 macrophages accelerate the resolution of inflammation but may also secrete too much TGF‐β to promote the proliferation and differentiation of lung fibroblasts and the progression of pulmonary fibrosis.^79^ Cell communication between fibroblasts and macrophages during lung injury and repair has been less studied. However, it is clear that blocking the recruitment of monocyte‐derived macrophages, promoting the apoptosis of M2 macrophages or inhibiting the polarization of M2 macrophages may be beneficial for the treatment of pulmonary fibrosis.^80^ Moreover, effective and timely repair of the lung epithelium can limit the inflammatory response, thereby preventing the progression of pulmonary fibrosis. It is believed that finding a way to repair the lung epithelium will aid the prevention and treatment of VILI, accelerate lung repair and improve the outcomes of lung injury patients.

## AUTHOR CONTRIBUTIONS

**Huan Liu**: Conceptualization; funding acquisition; software; writing—original draft; writing—review and editing. **Xuepeng Yang**: Writing—review and editing. **Ge Liu**: Writing—review and editing.

## CONFLICT OF INTEREST STATEMENT

The authors declare no conflict of interest.
